# Patient perceptions of virtual reality for pain relief in labor: A qualitative study

**DOI:** 10.3389/fpain.2022.1063751

**Published:** 2022-12-13

**Authors:** Melissa S. Wong, Kimberly D. Gregory, Brennan M. R. Spiegel, Carine Khalil

**Affiliations:** ^1^ Department of Obstetrics and Gynecology, Cedars-Sinai Medical Center, Los Angeles, CA, United States; ^2^ Division of Informatics, Cedars-Sinai Medical Center, Los Angeles, CA, United States; ^3^ Department of Obstetrics and Gynecology, David Geffen School of Medicine at UCLA, Los Angeles, CA, United States; ^4^ Department of Community Health Sciences, UCLA Fielding School of Public Health, Los Angeles, CA, United States; ^5^ Cedars-Sinai Center for Outcomes Research and Education (CS-CORE), Los Angeles, CA, United States; ^6^ Division of Digestive and Liver Diseases, Cedars-Sinai Medical Center, Los Angeles, CA, United States; ^7^ Division of Health Services Research, Cedars-Sinai Medical Center, Los Angeles, CA, United States; ^8^ Le Laboratoire Interdisciplinaire de Recherche Appliquée en Economie de la Santé (LIRAES) Lab, Paris Descartes University, Paris, France

**Keywords:** labor, pregnancy, obstetrics, pain, virtual reality, qualitative

## Abstract

**Introduction:**

Labor represents the most common reason for hospitalization, and most patients will use some form of pain management during their labor. While some studies have suggested that virtual reality (VR) may be an effective option for managing pain, more study is necessary to understand the patient experience of VR. The aim of this study is to characterize the effect of VR on patient perceptions of coping in labor and their descriptions of the VR experience.

**Methodology:**

A nested prospective, descriptive study within a randomized controlled trial of VR in laboring patients. We included nulliparous, term patients, having contractions at least every 5 min, a pain score on the Wong-Baker pain scale of 4–7, and who had been randomized to receive the 30 min virtual reality intervention in the trial. Subjects completed a childbirth self-efficacy inventory prior to the intervention. After the intervention, they completed a modified childbirth self-efficacy inventory related to VR and underwent a structured interview. Self-efficacy scores were compared using t-tests and qualitative, thematic analysis was performed using Dedoose.

**Results:**

Twenty-one subjects received the VR intervention. Twenty subjects completed the post-intervention survey and structured interview; one declined due to discomfort. Subjects noted a significant increase in perceived degree to which VR could improve their self-efficacy in managing pain during labor. Thematic analysis revealed that subjects described the VR experience as allowing them to connect with their breathing, feeling more relaxed, and being distracted from pain. In total, 70% believed VR reduced their pain, 60% felt it reduced their anxiety, and 100% would recommend VR availability for laboring patients.

**Conclusion:**

VR can improve patient self-efficacy for managing pain in labor. Future studies should focus on the content of the visualizations, optimized user experience and design, and effectiveness with ongoing exposure to VR content in labor.

## Introduction

Labor represents the most common reason for hospitalization in the United States, exceeding the next two diagnoses combined (1). Labor is also considered one of the most painful experiences humans undergo (2). Patients often attempt to mitigate this pain through the use of non-pharmacologic methods such as biofeedback, aromatherapy, acupuncture, and self-hypnosis.

Virtual reality (VR) therapy describes the use of a headset with a close-proximity stereoscopic screen to deliver images and videos which create an immersive experience intended to yield a response for the viewer. VR is believed to rely on a combination of distraction, time acceleration, and alterations to gate control of pain signals to reduce perception of nociceptive stimuli and pain (3). It has demonstrated benefits in both acute (e.g., procedure-related) and chronic pain (4, 5). We and others have previously demonstrated the effectiveness of VR in reducing pain in persons in labor (6–9). Our study was unique in that – unlike others – our visualization was specifically targeted towards laboring persons. In addition, following the intervention, we conducted structured interviews to better evaluate the patient's perspective on the use of VR and its impact on their pain perception.

The objective of this qualitative study is to characterize the effect of VR on patient perceptions of coping in labor and their descriptions of the VR experience.

## Methodology

### Study design and procedures

We conducted a prospective, descriptive study within an open-label randomized controlled trial to assess the effect of VR on pain management for pregnant persons in labor. Prior to initiating the trial, it was approved by the Cedars-Sinai Medical Center Institutional Review Board (Pro00050082) and the protocol registered with clinicaltrials.gov (NCT03437031). Enrollment began in March 2018 and completed in February 2019.

The details of the randomized controlled trial have been described in detail elsewhere (8). In brief, subjects were included if they were nulliparous, term, having regular contractions (defined as at least every 5 min), and with a pain score on the Wong-Baker pain scale between 4 and 7. In addition, subjects were required to be ≥18 years old, English-speaking, and able to give consent. Subjects were excluded if they had used any medications for pain relief prior to the intervention including oral or intravenous pain medications or neuraxial analgesia. Subjects were also excluded if they had a preterm gestation, pain not due to contractions, or pain scores outside of the intended range. Finally, subjects were excluded if the use of VR would not have been appropriate for them such as those at risk for seizures (preeclampsia with severe features, eclampsia, history of epilepsy, etc), sensitive to flashing light/motion, having a medical condition predisposing to nausea/dizziness, or with any injury to the eyes/face/neck or arms that would make the use of the hardware uncomfortable.

### Conduct of the survey and interview

**Figure 1:**
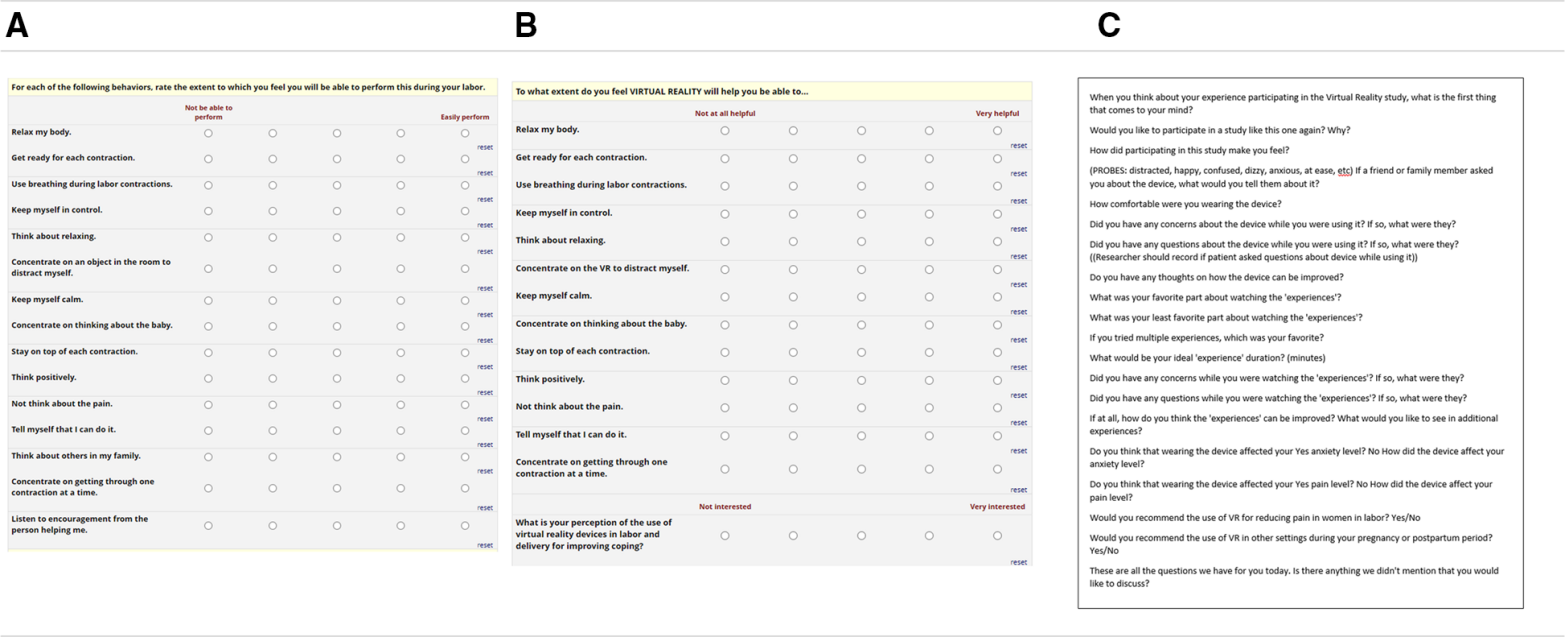
Data collection instruments. REDCAP surveys assessing Childbirth Self Efficacy Inventory (**A**), Modified Childbirth Self Efficacy Inventory for VR (**B**), and Guided interview questions (**C**).

Subjects were identified, screened, and consent was obtained. All subjects also completed a Childbirth Self Efficacy Inventory prior to the intervention (Figure 1A). The Childbirth Self Efficacy inventory is a validated, self-reporting tool to assess perceived confidence and coping for labor (10). Subjects were then randomized to either receive up to 30 min of VR or up to 30 min of no intervention. The specific virtual reality intervention was Labor Bliss, a program developed by appliedVR for the purpose of mitigating pain in patients in labor. Subjects were taken through the same three visualizations, each lasting 10 min: first, a tree which would expand and contract; second, a glowing campfire, and third, a beach scene with waves crashing into frame. Importantly, these visualizations were accompanied by audio guidance pertinent to patients in labor, e.g., “Contractions are waves of energy bringing your baby closer to you.”

Following the intervention, all patients who received VR were further evaluated with a structured interview and modified Childbirth Self-Efficacy inventory specifically asking whether VR would augment their self-efficacy in labor (Figure 1B). The subject completed both the pre-interview CSEI and the post-interview CSEI independently, entering their responses into a Redcap form on a tablet (11). The structured interview was conducted each time by a single investigator and responses were documented in real time using a Redcap form (Figure 1C). Questions that participants were asked included*: “when you think about your experience participating in the Virtual Reality study, what is the first thing that comes to your mind”; “how did participating in this study make you feel?”*; “*what was your favorite part about watching the experiences?”*; “*what about your least favorite part?”*; “*did you have any concerns while you were watching the experiences? If so, what were they?”*; “*do you have any thoughts on how the device can be improved?”* (Figure 1C).

### Thematic and statistical analysis

An inductive thematic analysis was used to examine the data collected during the interviews. The analyses were performed by an experienced researcher (C.K.) with formal training in qualitative methods using the Dedoose software package. The transcripts were carefully read multiple times to enable total immersion in the discussions. Throughout the reading, sentences and paragraphs were highlighted and coded. Hence, key labels were inductively identified in the unstructured data. After sorting, combining, and refining the generated codes and labels, a set of inductive themes and subthemes were defined and justified with verbatim quotes. Of note, thematic saturation was achieved after completing 15 interviews. Similar instances were found in the data after the 15^th^ interview. Afterwards, data summaries were presented to all research team members as part of peer debriefing to discuss the insights obtained from the interviews and to refine the qualitative analysis network.

Analysis of differences in the pre and post intervention self-efficacy scores was performed using two-tailed t-tests, assuming unequal variances. Statistical tests were performed using SPSS Statistics® version 24; *p* < 0.05 was used as threshold for statistical significance.

## Results

Twenty-one subjects received the VR intervention; nineteen were allocated to the control (no intervention) arm. The baseline mean CBSEI scores for the VR group were 105.1. The mean self-efficacy scores in the original Lowe trial were 103.1; this score corresponds to average self-efficacy (10). Following the intervention, 20 subjects completed the post-intervention survey and interview. One subject declined to complete the post-intervention survey and interview due to discomfort and a desire have her epidural immediately.

**Figure 2:**
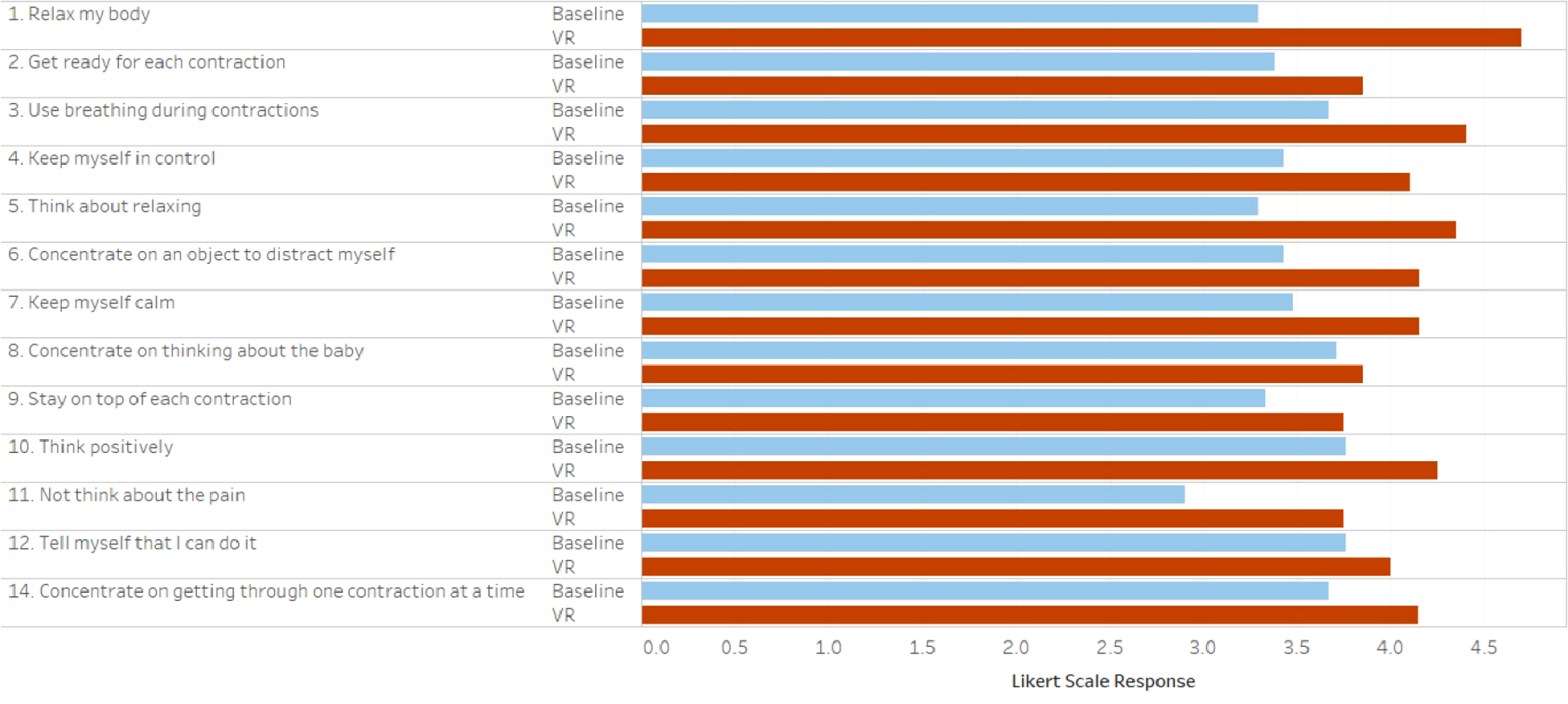
Baseline and post-VR childbirth self-efficacy indices. All individual values and adjused sum totals were statistically significantly improved in the VR group.

**Figure 3:**
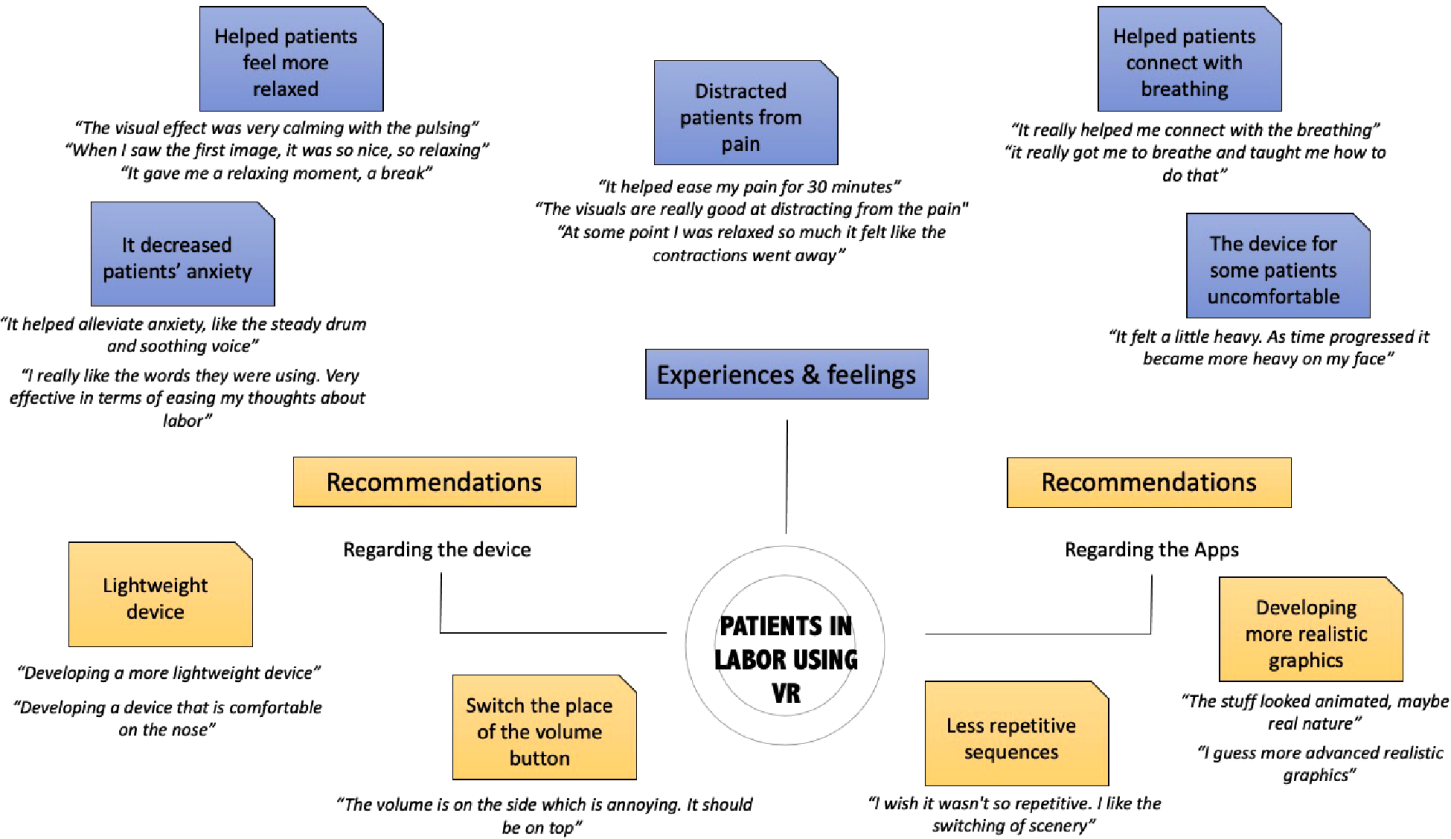
Qualitative mind map from guided interviews of pregnant persons who underwent VR in labor.

The patient's perceived degree to which VR would improve each CBSEI measure and their CBSEI score overall was significant (Figure 2), with respondents scoring 123.2 and demonstrating higher scores for each individual question (all *p* < 0.001). The qualitative interviews show that the use of VR was perceived as calming, relaxing, and distracting from pain. According to the patients, the use of VR helped them “*connect with the breathing”*, “*relax”*, and “*focus on something else”* while laboring (Table 1). It also created a distraction from the pain they were experiencing. Patients were mostly satisfied with the experience they had “*the experience was so beautiful”*, “*I really liked the messages about thinking of your contractions as waves of love”*. Several patients wanted to encourage other patients to use VR during labor or to at least “*try it before any pain medication”*. They appreciated the music, the pictures, graphics as well as the guiding messages, which helped them control the breathing and better handle the pain “*I liked that it reminds you to breathe and focus on the contractions as a pathway to meeting your baby”*. However, the use of the device was a somewhat uncomfortable for some patients: “*it felt a little heavy”, “as time progressed it became more heavy on my face”*. Hence, the development of a lightweight device, that “*has less weight on the nose”*, was suggested by many participants. Figure 3 highlights some of the main themes of the patients' experience with VR during labor.

**Table 1:** Patients’ experience of VR during labor – thematic analysis.

Patients’ experience with VR	Verbatim
Connect with breathing	*“It really helped me connect with the breathing”* *“it really got me to breathe and taught me how to do that”*
Feeling more relaxed	*“I'd never heard about anything like it. Relaxing. When I saw the first image it was so nice, so relaxing”* “*The visual effect was very calming with the pulsing”* *“It gave me a relaxing moment, a break”*
Being distracted from pain	*“It helped ease my pain for 30 min”* *“The visuals are really good at distracting from the pain”* *“At some point I was relaxed so much it felt like the contractions went away.*

Patients' recommendations included less repetition and technical improvement in both the graphics and device comfort. 70% believed VR reduced their pain, 60% felt it reduced their anxiety and 100% would recommend it for laboring women.

## Conclusions

The objective of this qualitative study was to characterize the effect of VR on patient perceptions of coping in labor and their descriptions of the VR experience. We demonstrated that VR was able to demonstrate significant improvements in patients' perceived sense of self-efficacy. The structured interviews provided a robust characterization of what aspects of the patient's experience of VR were most impactful as well as what the next steps would be in further developing a labor-specific VR experience.

### Context of this study

There have been few studies of the use of VR for pain relief in laboring patients. The first study of this application was limited to a 10-minutes intervention and utilized a crossover design (9). Our randomized controlled trial was only the second study of this kind and demonstrated a statistically significant reduction in pain compared to those who received no intervention (8).

### Strengths and limitations

To our knowledge, this is the first qualitative study of the use of VR for pain reduction for pregnant persons in labor. While our previous quantitative study was able to demonstrate objective reductions in pain scores, this study prioritizes the patient voice and experience over the quantitative changes in pain score. The participants validated the importance of the use of a pregnancy-specific visualization which has not previously been studied; most VR interventions have relied on generic relaxation experiences and non-specific musical background.

Our study is limited by its restriction to a single institution, a single VR headset and visualization and small sample size. However, an inductive thematic saturation was reached after the 15th interview as no new codes occurred in the data. The additional interviews that were performed confirmed that data saturation was reached.

### Future directions

VR remains vastly understudied in women's health and obstetrics in particular, despite reproductive age persons being technologically engaged and interested in complementary and alternative methods of patient management. Our study demonstrates that patients are interested in pregnancy-specific interventions and found the experience of VR positive. Improvements could be made to the visualizations and repetition as well as to the physical nature of the device (though this likely reflected that we were using a 1st generation device at the time).

Our next steps will be to further analyze the effect of VR on continuous maternal and fetal indicators, specifically maternal heart rate variability and fetal heart rate changes, as well as to evaluate impact on analgesic and anesthetic use. Ultimately, our goal is to optimize a pregnancy-specific experience and make continuous use of the headset available at the bedside during a patient's labor. In doing so, we anticipate being able to demonstrate ongoing effectiveness and potential benefits to reducing analgesic and anesthetic use.

## Data Availability

The datasets presented in this article are not readily available because they have been previously deidentified due to duration of consent for use. Requests to access the datasets should be directed to, wongmsx@cshs.org.
